# The protective role of cognitive reserve: an empirical study in mild cognitive impairment

**DOI:** 10.1186/s40359-024-01831-5

**Published:** 2024-06-07

**Authors:** Giulia Marselli, Francesca Favieri, Giuseppe Forte, Ilaria Corbo, Francesca Agostini, Angela Guarino, Maria Casagrande

**Affiliations:** 1 Department of Psychology, “Sapienza” University of Rome, Rome, Italy; 2 Department of Dynamic and Clinical Psychology and Health, “Sapienza” University of Rome, Via degli Apuli 1, Rome, 00184 Italy; 3 Save The Parents, APS, Rome, Italy

**Keywords:** Cognitive reserve, Mild cognitive impairment, Aging

## Abstract

**Background:**

Mild cognitive impairment (MCI) describes an aging profile characterized by a cognitive decline that is worse than expected in normal aging but less pervasive and critical than full-blown dementia. In the absence of an effective treatment strategy, it is important to identify factors that can protect against progression to dementia. In this field, it is hypothesized that one aspect that may be a protective factor against the neurotypical outcome of dementia is cognitive reserve (CR). Cognitive reserve is the ability to maintain cognitive functionality despite accumulating brain pathology.

**Objectives:**

The present study aimed to identify and analyze the differences in CR between healthy adults and patients with MCI. Specifically, it is hypothesized that (i) healthy older adult people have higher CR than older adult people diagnosed with MCI, and (II) CR could predict the classification of subjects into people with or without MCI.

**Methods:**

Two hundred forty-three adults (mean age = 60.4, SD = 7.4) participated in the present study and were classified into three groups based on Petersen’s MCI criteria: healthy controls (HC), amnestic MCI (aMCI), and non-amnestic MCI (naMCI). The Cognitive Reserve Index questionnaire (CRIq) was administered to assess the level of CR,

**Findings:**

Results showed that HC had significantly higher CR scores than participants diagnosed with aMCI and naMCI. Moreover, a binomial logistic regression suggested that low CR was a significant risk factor for the MCI diagnosis.

**Conclusions:**

The clinical picture that emerged from the results showed that lower CR could be considered a characteristic of pathological aging, such as MCI.Public significance statement, Since the brain attempts to cope with life-related changes or pathologies, it is fundamental for both clinicians and researchers to investigate further the factors that contribute to brain resilience. As an indirect expression of brain reserve, cognitive reserve may be both a marker and a predictor of adaptive aging.

## Background

The brain changes occurring with dementia can begin many years before the overt symptomatology, and they are usually expressed by the Mild Cognitive Impairment (MCI) phenotype [1–3]. The cognitive degeneration that characterizes MCI is worse than expected in typical aging but less pervasive and critical than full-blown dementia [2]. Moreover, unlike dementia syndromes, patients with MCI preserve independence in their activities of daily living [2]. Since MCI can represent a transitional phase between healthy aging and dementia syndromes [2], it is a condition of great clinical relevance, allowing clinicians and researchers to focus on approaches that could delay or counteract the conversion from MCI to dementia [4, 5]. This may be useful, especially in a framework characterized by the absence of effective treatments for dementia [6]. Accordingly, it is relevant to identify factors that can protect against the progression of cognitive decline from MCI to dementia, and current research is gradually shifting the attention from claimed dementia to early stages of cognitive decline, such as MCI.

In this frame, it is hypothesized that one of the protective factors against the neurotypical outcome of dementia is cognitive reserve (CR). In its most recent definition, CR has been described as a brain attribute enabling higher cognitive abilities despite significant life-related brain changes, injury, or disease [7]. It has been hypothesized that constant brain activity earlier in life helps counteract the pathological brain changes of aging, thereby delaying the onset of dementia symptoms. Operationally, cognitive reserve represents a latent construct that can be inferred from specific socio-behavioral indices [7].

The research topic of cognitive reserve in cognitive decline is quite timely, and the benefits of reserve for cognition in older adults have been demonstrated many times, highlighting an association with the dementia phenotype [8–10]. This evidence would justify the empirical observation that, in some cases, the levels of brain pathology do not match with clinical symptoms [11].

In other words, the clinical manifestation of cognitive impairment seems to be influenced by the level of cognitive reserve (Stern, 2009).

So, experiences related to education, work, and leisure activities would determine individual differences in the ability to cope with brain damage due to physiological aging or clinical conditions and, consequently, to maintain good functioning in daily life.

In particular, focusing on the role of cognitive reserve in pathological aging, some studies recognize a protective effect of CR by estimating that high levels of cognitive reserve reduce the risk of developing dementia by about 46% (Valenzuela & Sachdev, 2005).

Moreover, life experiences related to schooling, type of occupation, and leisure activities are also associated with cognitive performance in healthy adults and older adults (Corral et al., 2006; Farina et al., 2018). So, even in healthy aging, there seems to be a positive impact of CR on cognitive functioning (Opdebeeck et al., 2016).

Thus, CR represents a protective factor “across” neurodegenerative diseases: in patients with Parkinson’s Disease (PD), high levels of CR seem to be associated not only with reduced cognitive impairment but also with reduced motor impairment (Guzzetti et al., 2019). Concerning Alzheimer’s Disease (AD), it has been observed that higher levels of CR are associated with a later onset of symptomatology (Stern, 2012).

While the protective role of CR in clinical conditions of dementia is widely recognized, its role in preclinical conditions such as MCI is still controversial.

However, some studies have shown how high levels of CR are associated with a reduced risk of developing MCI [12–14]. Similarly, some studies suggested that CR is a risk factor for conversion from MCI to dementia (Allegri et al., 2010; Xu et al., 2019). This evidence has led several authors to propose cognitive reserve as a target for interventions to prevent MCI [5, 14].

Based on this evidence, one might hypothesize that CR may also be a marker to discriminate between healthy aging and early and less severe stages of cognitive impairment (e.g., MCI).

Accordingly, this study focused on the differences in cognitive reserve between individuals reporting healthy aging and those diagnosed with MCI. In the present research, we will be referring to cognitive reserve in general, even if we are measuring its proxy, since we do not assess brain status.

Therefore, the first aim of the present study was to identify and analyze the differences in CR proxy scores between healthy adults and patients with MCI. Specifically, it was hypothesized that healthy older adults would have a higher CR than older adults diagnosed with MCI, without any difference between MCI subtypes. Although this difference in CR has already been addressed and demonstrated in the scientific literature [13, 15], it seems appropriate to confirm these results and to investigate further this relationship, which defines the potential modulation of the CR on the classification of (1) people with or without MCI and (2) people with different subtypes of MCI (e.g., amnestic MCI and non-amnestic MCI). Since CR is generally associated with global functioning [13, 16], another aim of the study was to assess whether and how CR is associated with global cognitive functioning in the different conditions investigated (e.g., healthy adults, patients with amnestic MCI, and patients with non-amnestic MCI). Finally, we hypothesized that CR could predict the classification of subjects as having or not having MCI.

## Methods

### Participants

Two hundred forty-three adults (mean age = 60.4, SD = 7.4; age range = 50–88; 66% females) voluntarily participated in the current cross-sectional study (sample characteristics are shown in Tables 1 and 2). Inclusion criteria were as follows: age over 50 years, absence of psychiatric, neurological, and cerebrovascular disorders, head trauma, brain injury, or brain surgery, and a score in the Mini-Mental State Examination (MMSE) score greater than 23. The absence of the former pathologies was assessed during the anamnestic interview.

Thirty-seven participants were not included due to traumatic brain injuries (*n* = 14), psychiatric disease (i.e., bipolar disorder, borderline disorder, *n* = 7), MMSE < 23 (*n* = 6), epilepsy (*n* = 4), stroke (*n* = 3), multiple sclerosis (*n* = 3).

We conducted a power analysis using GPower in order to calculate the minimum sample size needed to detect the specified hypothetical effect size. This analysis revealed that we would need a sample size of at least 86 in each group to be confident (with a probability greater than 0.9) of detecting an effect size of δ ≥ 0.5, assuming a two-sided criterion for detection that allows for a maximum Type I error rate of a = 0.05 (Cohen, 1988).

.All participants were Italian, and data collection was carried out in the metropolitan area of Rome, Italy. Participants were recruited through announcements at the Health Psychology Laboratory at the Department of Dynamic and Clinical Psychology, and Health Studies of the “Sapienza” University of Rome and posters were placed in community centers like senior centers, gyms, businesses, etc. The timeframe of data collection goes from 2018 to 2023.

### Instruments

#### Anamnestic interview

A face-to-face interview collected general and medical information. Specifically, researchers gathered biographical information (age, marital status, education, occupation, etc.), lifestyle information (smoking habits, alcohol, and coffee consumption), medical conditions including mental health (e.g., history of psychiatric disorders, psychopathological diagnosis), and physical health (e.g., neurological diagnosis, brain injury, clinical condition).

#### Cognitive Reserve Index questionnaire

To assess the level of CR, the Cognitive Reserve Index questionnaire (CRIq) [17] was administered. The CRIq is a 20-item questionnaire that explores three different life domains through multiple questions about habits and life experiences: (i) education (e.g., school attendance and possible training courses), (ii) working activity (e.g., jobs held over the lifespan categorized by the level of responsibility and intellectual effort), (iii) and leisure time activities (e.g., enrichment activities such as attending theater, museums, or cinemas, reading). A score is given for each of the three domains, and a total score of CR is provided to determine each participant’s overall level of CR. According to Nucci et al. [17], the CRIq total score can be classified into five levels: low (score < 70), medium-low (70–84), medium (85–114), medium-high (115–130), high (> 130).

#### Psychological assessment

To assess trait anxiety was administered the State-Trait Anxiety Inventory - Y (STAI-Y) [Spielberg et al., 1983; Pedrabissi and Santinello, 1989]. To evaluate depression, two questionnaires were used: the Geriatric Depression Scale (GDS) [Kurlowicz and Greenberg, 1999; Chattat et al., 2001] and the Beck Depression Inventory (BDI) [Beck et al., 1961; Scilligo, 1983].

#### Neuropsychological assessment

To assess the global level of cognitive functioning, we adopted the Mini-Mental State Examination (MMSE) [18] and Raven’s Standard Progressive Matrices (RSPM) [19]. Moreover, we measured the level of autonomy in the activities of daily living with two questionnaires: Activities of Daily Living (ADL) [20] and Instrumental Activities of Daily Living (IADL) [21].

Cognitive domains for the diagnosis of MCI were assessed by a complete neuropsychological assessment, which included the evaluation of the following domains:

(i) Memory domain in both visuospatial and verbal components of memory.

To assess *verbal memory*, we adopted the Digit Span Forward test [22], the Rey Auditory Verbal Learning Test [23], and Babcock’s Tale [24]. These tasks allowed us to evaluate short-term, long-term, and semantic memory, respectively.

To assess *visuospatial memory*, we adopted the Immediate Visual Memory task (IVM) [25] and the Rey-Osterrieth Complex Figure (FRD) [26] to evaluate both short-term and long-term components.

(ii) The language domain was evaluated using a Sentence Construction test [23] and the tests of verbal fluency in the phonemic (PF) and semantic (SF) categories [27].
(iii) Attention was assessed using the Attentional Matrices [24] and the A form of the Trail Making Test (TMT-A) [28].
(iv) Visuospatial functioning was measured through the Clock Drawing Test (CDT) [29], the copy of the Rey-Osterrieth Complex Fig. [26], and through a task requiring participants to copy drawings with and without landmarks (CD, CDL) [25].
(v) Executive functions were assessed using the Digit Span Backward test [22], the B form of the Trail Making Test (TMT-B) [28].

All scores of cognitive tasks are adjusted for age and education according to their Italian validation.

#### Procedure

This study was conducted according to the Declaration of Helsinki and was approved by the Institutional Review Board of the Department of Psychology, Sapienza University of Rome (protocol number: 0001063). Before the neuropsychological assessment, participants signed an informed consent form, which indicated that they were willing to participate and understood the aims of the study and procedures. The assessment began with an anamnestic interview, and then the neuropsychological tests and the various questionnaires were administered in a randomized and counterbalanced order. The evaluation lasted approximately three hours, and to not excessively overburden the participants, the procedure was split into two parts, separated by a half-hour interval. In some cases (e.g., fatigue or other needs of the participants), the evaluation was completed on two different days within a week.

#### Group classification

According to Petersen’s criteria [2], participants were classified as healthy controls (*N* = 135; 63% female, mean age = 59.05, SD = 6.22, mean years of education = 15.93, SD = 3.45) and participants with MCI (*N* = 108; 69% female, mean age = 62.17, SD = 8,05, mean years of education = 13.78, SD = 3.97). According to Petersen’s criteria [2] the cut-off was set at minus 1.5 SD. Participants with MCI were classified into two groups: amnestic MCI (aMCI; *N* = 53, 66% female, mean age = 64.83, SD = 8.40, mean years of education = 13.25, SD = 3.94) and non-amnestic MCI (naMCI; *N* = 55, 73% female, mean age = 59.51, SD = 7.71, mean years of education years = 14.31, SD = 4.00). The former group had an impairment in the memory domain, while the latter group had preserved memory abilities but impairments in other cognitive domains (such as executive functions, language, etc.).

### Data Analysis

A series of ANOVAs were conducted considering the Group (HC, aMCI, naMCI) as the independent variable and some demographic (e.g., age, education), cognitive (MMSE and RSPM), psychological (GDS, BDI, STAI-Y), functional (ADL and IADL) characteristics, and lifestyle habits (e.g., cigarettes, coffee, or alcohol consumption, number of children, and number of cohabitants) as dependent variables.

In addition, the χ2 test was used to evaluate whether the three groups differed on categorical variables (e.g., sex and marital status).

To analyze the differences in CR among the three groups (HC, aMCI, naMCI), a series of ANCOVAs were conducted, considering the Group as the independent variable, the CR scores (CRI-Total, CRI-Ed, CRI-WA, CRI-LT) as the dependent variables, and age as a covariate. The Tukey test was chosen for post hoc analyses. Linear correlations (Pearson’r) were performed to examine the relationships between CR (CRI-Total, CRI-Ed, CRI-WA, CRI-LT) and global cognitive functioning (MMSE and RSPM).

Binomial logistic regression was performed, using CRIq total score and age as predictors and diagnosis (HC or MCI) as dependent variables. Moreover, two linear regressions were performed to test whether CRIq scores could predict the level of impairment, measured as the number of impaired cognitive tasks.

## Results

Table 1 shows the results of the ANOVAs and post-hoc analyses carried out between the groups (i.e., HC, aMCI, naMCI).

Relatively to demographic and cognitive variables, significant differences were found in age (F = 13.2, *p* < 0.001), years of education (F = 11.3, *p* < 0.001), MMSE scores (F = 21.3, *p* < 0.001), and RSPM scores (F = 19.8, *p* < 0.001). Specifically, HC and naMCI were significantly younger than the aMCI group (HC = 59.1 *v.s* aMCI = 64.8; naMCI = 59.5 vs. aMCI = 64.8. Both p-values are < 0.001).

HC group reported significantly higher years of education than both the naMCI (15.93 vs. 14.31; *p* = 0.018) and aMCI (15.93 vs. 13.25; *p* < 0.001). Similarly, RSPM scores were higher in healthy participants than in both aMCI (37.2 vs. 31.1; *p* < 0.001) and naMCI (37.2 vs. 32.3; p<. 001) groups.

Moreover, considering the global level of cognitive functioning, MMSE scores were lower in individuals with aMCI than in individuals with naMCI (28.2 vs. 28.8; *p* = 0.037) and healthy controls (28.2 vs. 29.4; *p* < 0.001); these latter groups also differed (naMCI = 28.8 vs. HC = 29.4; *p* = 0.002).

No differences emerged between groups for lifestyle habits and psychological characteristics (anxiety and depression).

Finally, the Chi-square test did not show significant differences in the gender distribution (χ2 = 2.55, *p* = 0.63) and marital status (χ2 = 13.7, *p* = 0.18).

**Table 1:** Mean (± SD) of demographic variables according to the diagnosis

	HC (*N* = 135)	aMCI (*N* = 53)	naMCI (*N* = 55)	df	F	*p*	pη^2^	Significant comparisons
Age	59.1 (6.22)	64.8 (8.40)	59.5 (7.71)	2, 240	13.2	< 0.001	0.099	A, C
Years of education	15.93 (3.45)	13.25 (3.94)	14.31 (4.00)	2, 240	11.3	< 0.001	0.086	A, B
ADL	5.95 (0.52)	5.92 (0.27)	5.89 (0.81)	2, 240	< 1	n.s.	0.002	
IADL	7.89 (0.74)	7.79 (0.63)	7.80 (1.11)	2, 240	< 1	n.s.	0.003	
MMSE	29.4 (0.90)	28.2 (1.56)	28.8 (1.14)	2, 240	21.3	< 0.001	0.150	A, B, C
RSPM	37.32 (6.00)	31.15 (8.69)	32.27 (7.26)	2, 240	19.8	< 0.001	0.142	A, B
Cigarettes/day	3.03 (6.56)	3.13 (6.03)	2.95 (6.89)	2, 239	< 1	n.s.	0	
Coffee/day	2.29 (1.94)	2.33 (1.27)	2.51 (1.43)	2, 238	< 1	n.s.	0.003	
Alcohol/week	3.11 (5.06)	4.50 (5.72)	5.10 (14.00)	2, 239	1.39	n.s.	0.012	
N. of children	1.91 (0.93)	1.60 (1.01)	1.80 (0.97)	2, 239	2.05	n.s.	0.017	
N. of cohabitants	1.70 (1.44)	1.48 (1.32)	1.71 (1.31)	2, 238	< 1	n.s.	0.004	
BDI	7.16 (7.33)	9.06 (9.18)	7.28 (8.22)	2, 237	1.13	n.s.	0.009	
GDS	2.72 (2.73)	3.42 (3.25)	3.0 (3.16)	2, 239	1.06	n.s.	0.009	
STAI-Y	37.97 (9.62)	39.71 (10.7)	38.42 (10.39)	2, 236	0.57	n.s.	0.005	

Significant comparisons: A = HC vs. aMCI - B = HC v.s naMCI - C = aMCI vs. naMCI

*Abbreviations: HC = healthy controls; aMCI: amnestic mild cognitive impairment; naMCI = non-amnestic mild cognitive impairment; ADL: Activities of Daily Living; IADL: Instrumental Activities of Daily Living; MMSE: Mini-Mental State Examination; RSPM: Raven’s Standard Progressive Matrices; BDI: Beck Depression Inventory; GDS: Geriatric Depression Scale; STAI-Y: State-trait anxiety inventory*

### Cognitive reserve according to the MCI diagnosis

Table 2 shows the distribution of our sample according to the MCI diagnosis and the level of CR. The Chi-square test showed significant differences in the CRI scores distribution across groups (χ2 = 14.73, *p* = 0.022). In particular, there was no significant difference across groups between the high and the medium-high scores (χ2 = 2.75, *p* = 0.25) since the significant difference was between the high and medium scores (χ2 = 9.68, *p* = 0.008). Moreover, aMCI vs. naMCI distribution did not differ significantly (χ2 = 0.325, *p* = 0.85), while naMCI vs. HC partially differed (χ2 = 5.92, *p* = 0.52) and aMCI vs. HC significantly differed (χ2 = 7.83, *p* = 0.02).

**Table 2:** Breakdown of participants into CRI levels described by Nucci et al. [17]

Diagnosis	High	Medium – high	Medium	Medium – low
**HC** (*N* = 135)	63%	23%	14%	0%
**aMCI** (*N* = 53)	42%	28%	28%	2%
**naMCI** (*N* = 55)	45%	24%	27%	4%

Abbreviations: HC = healthy controls; aMCI: amnestic mild cognitive impairment; naMCI = non-amnestic mild cognitive impairment

Table 3 shows the level of CR of the three groups of participants, both for the total CRI and its three components (CRI-Ed, CRI-WA, and CRI-LT). ANCOVAs were conducted, considering the Group as the independent variable, the CR scores (CRI-Total, CRI-Ed, CRI-WA, CRI-LT) as the dependent variables, and age as a covariate. ANCOVAs showed significant differences in the CRIq total score (F = 8.98, *p* = 0.036) and CRI-Education (F = 5.45, *p* = 0.022), while in the CRI-Work Activity (F = 2.39, *p* = 0.12) and CRI-Leisure Time (F = 2.66, *p* = 0.10) no differences emerge when considering age as a covariate. In addition, considering ANOVAs, significant differences emerge in all indices of CRIq. The CRIq total score (F = 14.77, *p* < 0.001) was significantly higher in the HC group compared to aMCI (136.7 vs. 124.2; *p* < 0.001) and naMCI (136.7 vs. 124.6; *p* < 0.001) groups. CRI - Education (F = 4.56, *p* = 0.011) was significantly lower in the aMCI group compared to HC (98.2 vs. 106.1; *p* = 0.011) and naMCI (98.2 vs. 106.0; *p* = 0.041) groups. Moreover, CRI- Work Activity (F = 9.61, *p* < 0.001) was significantly higher in the HC group compared to aMCI (130.9 vs. 120.8; *p* < 0.01) and naMCI (130.9 vs. 118.2; *p* < 0.001) groups. Finally, CRI- Leisure Time (F = 6.72, *p* = 0.001) was also significantly higher in the HC group compared to the naMCI group (146.1 vs. 130.5; *p* < 0.001).

**Table 3:** Mean (± SD) of CR scores in the three groups of participants and ANCOVA results

	HC (*N* = 135)	aMCI (*N* = 53)	naMCI (*N* = 55)	df	F	*p*	pη^2^	Significant comparisons
CRI Total	136.7 (19.4)	124.6 (19.8)	124.2 (20.4)	2, 1, 239	8.98	0.003	0.036	A, B
CRI-Ed	106.1 (21.7)	98.2 (20.5)	106.0 (21.4)	2, 1, 239	5.45	0.02	0.022	A, C
CRI-WA	130.9 (21.1)	120.8 (22.1)	118.2 (21.9)	2, 1, 239	2.39	0.12	0.010	A, B
CRI-LT	146.1 (30.5)	136.4 (31.5)	130.5 (26.7)	2, 1, 239	2.66	0.10	0.011	A, B

Significant comparisons: A = HC vs. aMCI - B = HC vs. naMCI - C = aMCI vs. naMCI

Abbreviations: HC = healthy controls; aMCI: amnestic mild cognitive impairment; naMCI = non-amnestic mild cognitive impairment; CRI-Ed: CRI-Education; CRI-WA: CRI-Working Activity; CRI-LT: CRI-Leisure Time

### Correlations among variables

Table 4 reports the correlations between CRI indices and cognitive performance in the HC and MCI groups.

No significant correlations between any CR score and global cognitive functioning are shown in healthy controls, whereas in people with MCI, the CRI-Ed and CRI Total were positively correlated with both MMSE and RSPM measures.

**Table 4:** Correlation matrix between cognitive functioning and CR scores in HC and MCI groups

	HC				MCI			
	CRI-Ed	CRI-WA	CRI-LT	CRI Total	CRI-Ed	CRI-WA	CRI-LT	CRI Total
MMSE	0.02	-0.08	0.04	0.01	0.25**	0.15	0.13	0.27**
RSPM	-0.12	0.16	0.17	0.13	0.35***	0.12	0.10	0.29**
DSF	-0.10	0.05	0.01	-0.02	0.13	0.06	0.10	0.16
RAVLT-I	0.21*	-0.01	-0.07	0.05	0.19	-0.02	-0.10	0.01
RAVLT-D	0.11	0.04	-0.04	0.05	0.20*	0.02	-0.09	0.05
Babcock	0.17*	-0.07	-0.12	-0.03	0.15	-0.01	-0.05	0.04
IVM	0.18*	-0.07	-0.10	-0.01	-0.10	0.02	0.21*	0.10
FRD	-0.08	0.28**	0.10	0.16	0.16	0.04	-0.06	0.06
PF	0.06	0.18*	0.18*	0.24**	0.17	0.06	0.22*	0.25**
SF	-0.12	0.15	0.19*	0.15	0.10	0.14	0.15	0.21*
SC	-0.07	0.07	0.10	0.07	0.01	0.13	0.12	0.15
AM	-0.04	0.08	0.09	0.08	0.25*	0.09	-0.05	0.14
TMT-A	0.16	-0.09	0.01	0.04	-0.22*	-0.08	-0.05	-0.18
CDT	0.08	-0.19*	-0.17*	-0.17*	-0.18	-0.14	-0.07	-0.20*
FRI	-0.17	0.29**	0.24**	0.23*	0.19	0.01	0.04	0.13
CD	0.10	0.01	-0.04	0.03	0.36**	0.22*	0.06	0.31**
CDL	0.06	-0.20*	-0.20*	-0.21*	0.14	-0.19	-0.13	-0.11
DSB	0.08	0.07	0.05	0.10	0.15	0.16	0.02	0.16
TMT-B	0.07	-0.17	-0.07	-0.09	-0.21*	-0.11	-0.08	-0.21*

* *p* < 0.05, ** *p* < 0.01, ***p < 0.001

Abbreviations: CRI-Ed: CRI-Education; CRI-WA: CRI-Working Activity; CRI-LT: CRI-Leisure Time; MMSE: Mini-Mental State Examination; RSPM: Raven’s Standard Progressive Matrices; DSF: Digit Span Forward test, RAVLT-I: Rey Auditory Verbal Learning Test Immediate recall; RAVLT-D: Rey Auditory Verbal Learning Test Delayed recall; IVM: Immediate Visual Memory task; FRD: Rey-Osterrieth Complex Figure; SC: Sentence Construction test; PF: phonemic fluency; SF: semantic fluency; AM: Attentional Matrices; TMT-A: Trail Making Test-A; CDT: Clock Drawing Test; FRI: copy of the Rey-Osterrieth Complex figure; CD: copy drawings without landmarks; CDL: copy drawings with landmarks; DSB: Digit Span Backward test; TMT-B: Trail Making Test-B.

### Binomial logistic regression

Binomial logistic regression was used to determine whether CRIq score could predict a diagnosis of MCI. CRIq score and age were set as predictors, while the diagnosis (HC or MCI) was the dependent variable. The MCI subtypes were combined for regressions since they were not sufficiently numerous to draw definite conclusions. Moreover, our focus was on the diagnosis of MCI in general, regardless of the cognitive domains.

The results of the binomial logistic regression were significant (OR = 1.038, p = < 0.001), indicating that individuals with higher CRIq scores had a higher chance of being considered healthy.

### Linear regressions

Two linear regressions were performed to test whether CRIq scores could predict the level of impairment, measured as the number of impaired cognitive tasks (below 1.5 SD from the mean). The number of impaired cognitive tasks according to the diagnosis is described in Table 5.

In the first linear regression, global CRIq score and age were set as the predictors, while the number of impaired tasks was the dependent variable. This model was significant (R^2^ = 0.07, *p* < 0.001), and both Age (*p* = 0.001) and CRIq (*p* = 0.02) can predict cognitive impairment. These results are shown in Table 6.

In order to evaluate the role of the single dimensions of CRI (Ed, WA, LT), we tested a second linear regression in which the three dimensions of CRI and age were set as predictors, while the number of impaired tasks was the dependent variable. This model was significant (R^2^ = 0.08, *p* < 0.001) and showed that Age (*p* = 0.01) and CRI-Education (*p* = 0.001) significantly predicted the subject’s performance, while CRI-WA and CRI-LT (*p* > 0.05) do not seem to be significant variables. These results are shown in Table 7.

**Fig. 1:**
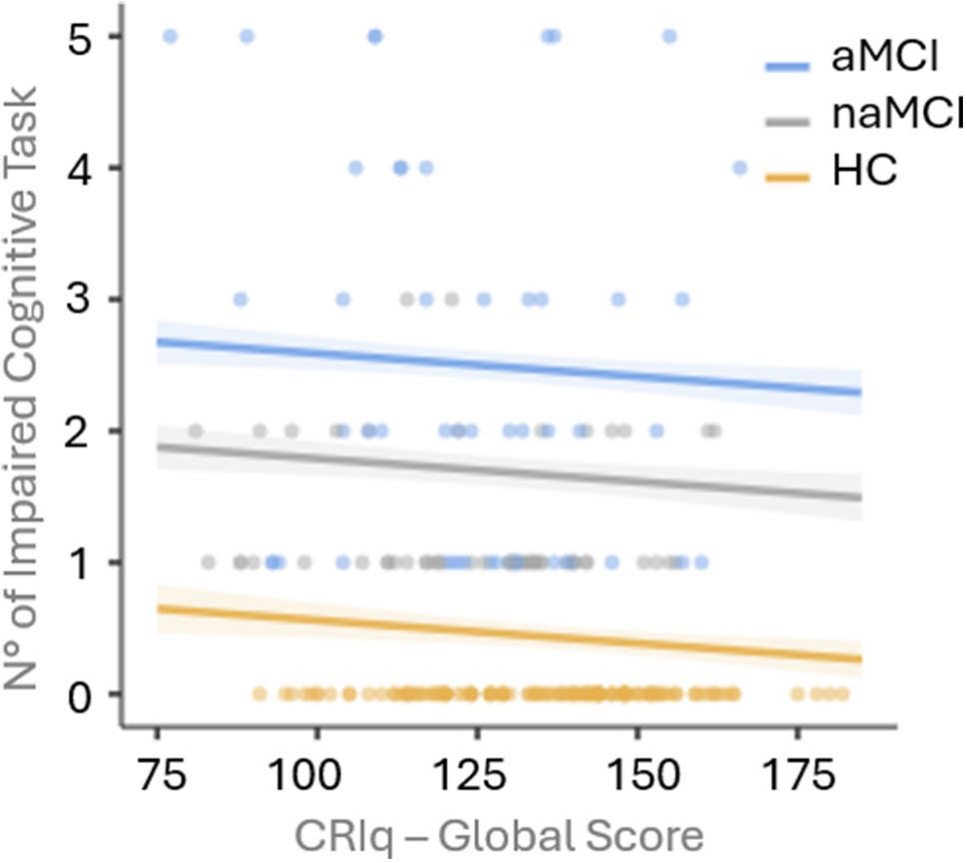
Observed CRIq global score in relation to the number of impaired cognitive tests, divided by group. Abbreviations: aMCI: amnestic Mild Cognitive Impairment; naMCI: non amnestic Mild Cognitive Impairment; HC: Healthy Control

Figure 1 shows observed CRIq global score in relation to the number of impaired cognitive tests, divided by group (aMCI, naMCI, HC).

**Table 5:** Impaired cognitive performance according to the diagnosis

	Mean	SD
MCI	2.4	1.9
HC	0.5	0.7

**Table 6:** Linear regression (global CRIq score and age as predictors)

Predictors	B	SE	t	CI (95%)	*p*
CRIq	-0.01	0.005	-2.28	-0.02/-0.001	0.02
Age	0.06	0.01	3.88	0.03/0.08	< 0.001

**Table 7:** Linear regression (CRI sub-scores and age as predictors)

Predictors	B	SE	t	CI (95%)	*p*
CRI-Ed	-0.01	0.005	-2.65	-0.02/-0.003	0.009
CRI-WA	-7.62	0.006	-0.13	-0.01/0.01	0.90
CRI-LT	-0.007	0.004	-1.56	-0.016/0.002	0.12
Age	0.06	0.01	4.03	-1.9/2.7	< 0.001

## Discussion

The present cross-sectional study examines the field of aging and aims to identify and analyze the differences in CR between healthy adults and patients with MCI. Seneca stated that “old age is an incurable disease”. However, this sentence does not take into account the concept of “successful aging” [30]. According to the positive aging perspective, our findings confirm that improving some lifestyle aspects could represent a personal defense to counteract dementia. Our results underline that CR may be promising to discriminate between healthy aging and mild cognitive impairment. The clinical picture in the present study suggested that lower CR could be considered a characteristic of pathological aging, such as MCI. From this perspective, lower CR increases the possibility of pathological aging. Since high CR allows responding flexibly to the physiological cognitive decline due to aging or neurodegeneration [31], our results can support the theory that a higher CR, throughout life and influenced by life experiences, increases cognitive flexibility and could be a characteristic that allows to delay the onset of dementia [11, 32]. Moreover, we further confirm that CR can also be considered one of the protective factors for global cognition in the early stage of cognitive decline. However, mild cognitive impairment has often been categorized as a clinical condition that includes many subtypes. Our study considered the amnestic and non-amnestic subtypes and found differences in CR in these groups. The inclusion of older adults with both aMCI and naMCI in the present study appears relevant because studies analyzing the relationship between CR and MCI usually tend to focus on the aMCI group [33–35], leaving out a significant proportion of individuals with MCI who are impaired in non-memory domains.

Our results suggest that CR may have been utilized as a compensatory mechanism against cognitive decline [11, 36]. These findings are consistent with previous evidence highlighting that a higher CR is associated with a higher global cognitive performance in older adults [32, 37] and in people with MCI [12–14]. These results confirm the well-established positive association between education and various cognitive functions, including memory, attention, and executive function in older adults [38–40], as well as on occupational attainment as a protective factor against cognitive decline in healthy older adults and progression from MCI to AD [41–43].

Interestingly, the correlation analysis highlighted that CR was significantly and positively correlated with global cognitive functioning only in participants with MCI.

Other authors have already demonstrated that people with high CR who develop MCI tend to present the onset of this clinical picture at a later age than individuals with low CR [13]. Our results go further and show that the relationship between CR and global cognitive pattern only emerged in people with MCI rather than in healthy individuals. To interpret this finding, a compensatory role of CR can be invoked. Neural compensation refers to the ability of individuals with brain pathology to use brain structures or networks, and therefore cognitive strategies, that are not typically used by individuals with intact brains to compensate for their damage [44]. It is important to note that this compensation is not always successful and may not fully restore cognitive function. Cognitive Reserve theory suggests that the brain copes with pathology or brain changes by utilizing preexisting cognitive processing approaches or compensatory mechanisms. According to this interpretation, individuals with mild cognitive impairment (MCI) may engage brain structures or cognitive strategies not typically utilized by those with normal brain function. The results of our study support this notion. Indeed, in our results, CR is solely associated with global cognitive functioning in participants with MCI since healthy individuals do not need to recruit this alternative network to compensate for age-related neural changes. In this case, the compensatory mechanisms implemented by participants with MCI are associated with certain maintenance of functioning, as opposed to improved functions.

Another important finding that emerged from our results is that CR can predict the diagnosis of MCI; in fact, lower CR is associated with a diagnosis of MCI. This finding is particularly relevant considering that no individuals with low CR are in our sample. People with high CR have been shown to have a significantly reduced risk of developing a pathological condition such as MCI [13, 14]. In addition, people with higher CR are more likely to reverse from MCI to normal cognition than to progress to dementia [15]. This interpretation suggests that CR can prevent or minimize cognitive decline. However, there is an alternative hypothesis: CR could act as a moderator between brain changes and clinical outcomes [11]. In patients with Alzheimer’s disease, there is evidence that higher CR is associated with more severe underlying pathology [45]. Indeed, patients with higher CR require more brain deterioration (such as reduced cortical thickness) before clinical symptoms of Alzheimer’s disease become apparent [45]. According to this latter hypothesis, the same pattern could occur in patients with MCI: those with higher CR may still present the diagnostic and neural conditions of MCI but with no or fewer observable clinical and behavioral effects. In this context, it would also be interesting to evaluate how cognitive reserve is related to better brain processes and more efficient physiological and autonomic functions [46, 47], as well as to psychological dimensions or adaptive coping strategies that improve behavioral outcomes [48].

Moreover, the results of the regressions showed that both age and the global level of cognitive reserve contribute significantly to explaining the overall level of cognitive impairment, defined as the number of cognitive domains in which participants perform below the normal range (e.g., 1.5 SD below the mean). In particular, among the various subscales of the CRIq, the subscale related to education (e.g., CRI-Education) appears to contribute uniquely to predict the level of cognitive impairment. This score is constituted of the years of education plus possible training courses, and this is the benefit of using this score instead of the years of education solely. These findings suggest that educational attainment (referring to both years of schooling and the level of education attained) is one of the factors that can best predict cognitive functioning in aging, in line with the results of a recent review [49]. On the one hand, this could be because investing time in learning and education would lead to better occupational outcomes and, on the other hand, it could “prepare” individuals to face performance tasks, such as the neuropsychological tasks proposed in the present study [50].

According to the findings highlighted by this study, some strengths and limitations should be defined. The main strength lies in the characteristics of the participants who were in the early stage of cognitive decline and the comprehensive measurement of CR, although the small sample size and the relatively young age of our sample can limit the generalizability of our results. Moreover, the cross-sectional, observational design of the study precluded the assessment of age-related changes in cognitive functioning and the role of CR as a mediator of these changes, preventing an inference of causality that a longitudinal study could have provided. Another limitation is represented by the fact that, according to the latest CR definition [7], the CRIq does not assess “the level of CR” but rather “Proxies for the cognitive reserve”.

An additional limitation of this study is that it focuses solely on cognitive aspects and does not consider biological factors such as APOE ε4. This omission could impact the severity and trajectory of Mild Cognitive Impairment (Wang et al., 2019; Albert et al., 2014).

Furthermore, it might be interesting to evaluate the relationship of CR not only with global cognitive functioning (e.g., MMSE) but also with specific cognitive functions that are impaired in MCI, such as simple and higher executive functions, attentional processes and their interaction with complex executive functions, or more complex memory functions [51–58]. The use of MMSE as a screening tool in healthy and MCI populations may present limitations due to its poor sensitivity in discriminating between these two groups. However, MMSE can effectively distinguish individuals with a diagnosis of dementia, which was an exclusion criterion for this study. Diagnostic outcomes were not based on the scores of this specific test.

Finally, our sample included only three participants in the medium-low level of CR [17] and no participants in the low level. Future research should include these levels in their sample in order to analyze how CR may affect pathological aging in this population.

### Conclusions and future implications

Despite the above-mentioned limitations, this study suggests that CR is associated with a more “successful aging” process.

The prevention and treatment of dementia is one of the most important challenges of our time. The possibility of a connection between certain life experiences and the development of dementia-like syndromes represents an important field of research. The gradual aging of the population is leading and will lead over the years to a steady increase in cases of dementia, especially Alzheimer’s dementia, for which there are no truly effective treatments [59].

Similarly, pharmacologic interventions in aMCI have not provided beneficial effects on the rate of conversion to dementia [60]. Cognitive training in patients with MCI has only shown positive short-term effects on memory functions [61]. These negative results could be due to the difficulty of diagnosing MCI [62]: in fact, the diagnostic criteria for MCI are very heterogeneous, and studying the results of interventions can prove dangerous as it may lead to contradictory results. Cognitive reserve might represent an aspect to be included in the diagnosis of MCI. Indeed, despite the clinical importance of cognitive reserve, a clinically useful diagnostic biomarker of brain changes underlying cognitive reserve in MCI is not yet available. Finding this biomarker could allow clinicians to diagnose MCI more effectively and researchers to develop more strategic interventions.

CR is an important factor to consider when studying “successful aging”, and several authors have suggested the possibility of developing and implementing preventive interventions targeting this construct [5]. CR is modifiable over the years and does not represent a static and unchanging dimension, especially in its more dynamic components, such as social and cognitively stimulating activities [5, 63].

Cognitive reserve, in its different expressions (schooling, work activity, leisure activities), represents an important factor to be considered in order to promote a healthy aging pathway and reduce the risk of developing clinical pictures typical of pathological aging, such as MCI [5, 63].

Further studies are needed to understand the concept of CR better and to identify optimal interventions that could promote CR and prevent dementia-like syndromes. Studies could combine several interventions, including exercise, cognitive stimulation, and social stimulation.

It would also be useful to replicate our findings in a longitudinal study and to assess whether and how the relationship between cognitive decline and CR is influenced by other psychological (e.g., depression, anxiety, alexithymia) and behavioral (e.g., sleep quality, diet, exercise) variables that were not assessed in this study.

Moreover, more studies are needed to clarify the concept of “reverse causation”, which suggests that individuals with a cognitive profile below the norm may have lower cognitive reserve. While our study interpreted the results in the opposite direction, a longitudinal study would be beneficial in providing further insight into this area.

As the brain attempts to cope with brain changes or pathology, it is fundamental for both clinicians and researchers to investigate further the factors that contribute to its resilience.

However, the study of MCI needs to be better systematized and deserves more attention due to its still evolving definition and multiple trends [62].

## Data Availability

The raw data supporting the conclusions of this article will be made available by the authors, without undue reservation.

## Abbreviations

ADL: Activities of Daily Living
aMCI: amnestic mild cognitive impairment
BDI: Beck Depression Inventory
CD: Copy drawings without landmarks
CDL: Copy drawings with landmarks
CDT: Clock Drawing Test
CR: Cognitive reserve
CRIq: Cognitive Reserve Index questionnaire
FRD: Rey-Osterrieth Complex Figure
GDS: Geriatric Depression Scale
IADL: Instrumental Activities of Daily Living
IVM: Immediate Visual Memory task
MCI: Mild cognitive impairment
MMSE: Mini-Mental State Examination
naMCI: Non-amnestic mild cognitive impairment
PF: Verbal fluency in the phonemic categories
RSPM: Raven’s Standard Progressive Matrices
SF: Verbal fluency in the semantic categories
STAI-Y: State-Trait Anxiety Inventory
TMT: Trail Making Test
